# Self-regulation and conflict goals management capabilities of ecosystem entrepreneurs: a case study of Haier ecosystem

**DOI:** 10.3389/fpsyg.2024.1384303

**Published:** 2024-05-21

**Authors:** Wenting Qin, Song Zhang, Baozhou Lu

**Affiliations:** ^1^Business School, Qingdao University, Qingdao, Shandong, China; ^2^School of Quality and Standardization, Qingdao University, Qingdao, Shandong, China; ^3^Management College, Ocean University of China, Qingdao, Shandong, China

**Keywords:** ecosystem entrepreneur, hub firm, new venture, conflict goals management capability, self-regulation

## Abstract

The inherent dual roles of “follower” and “leader” among ecosystem entrepreneurs inevitably introduce challenges in managing conflicting dependent and independent goals. Ecosystem entrepreneurs’ capabilities in conflict goals management directly influence new venture survival and development. This single-case qualitative study explores how ecosystem entrepreneurs develop conflict goals management capabilities through self-regulation, which is not only a unique practical challenge in ecosystem entrepreneurship, but also a cutting-edge topic in current theoretical research. Through research of entrepreneurs in Haier Entrepreneurship Ecosystem, the paper finds: (1) strategic corresponding and mechanism adapting emerge as the two trigger factors enabling ecosystem entrepreneurs to recognize the equilibrium or disequilibrium between conflicting goals; (2) by leveraging self-control, grit, and metacognition, ecosystem entrepreneurs construct decoupling mechanisms for antagonistic goal recognition and coupling mechanisms for synergistic goal recognition; (3) ecosystem entrepreneurs enhance their conflict goals management capabilities by developing both segregative and synergistic management capabilities. Furthermore, this research explores the self-regulation process underlying ecosystem entrepreneurs’ conflict goals management behaviors, including environmental interaction perception, conflict goals analysis, and delineation of goal relationships. Findings provide insights for ecosystem entrepreneurs on improving their conflict goals management capabilities through self-assessment and skill development.

## 1 Introduction

Ecosystem entrepreneurship has emerged as a trend among technology entrepreneurs (Muegge, 2013; Jiang et al., 2023). Ecosystem entrepreneurs uniquely face conflicting goals. On one hand, they must meet the hub firm’s objectives to secure entrepreneurial resources and opportunities. On the other hand, they must continuously pursue their entrepreneurial goals to gain independent growth possibilities outside the ecosystem. In practice, the new venture’s and ecosystem’s performance goals often differ in scope and time horizon. For instance, the hub firm may sacrifice short-term profits for greater market share, while short-term profits directly impact new venture survival. When Haier ecosystem incubator Youzu.com was suddenly required to fully separate from Haier Group, its entrepreneurs were shocked and overwhelmed. Such scenarios underscore that building capabilities to manage conflicting goals is an inevitable and critical challenge for ecosystem entrepreneurs.

Existing research presents differing perspectives on building capabilities for managing conflicting goals. In order to manage conflict goals in cognition and behavior with the new ventures, the hub firm establish platform leadership to improve conflict goals management capabilities (Levitt et al., 2024). Some scholars consider the hub firm and the new ventures as a whole, emphasizing shared innovation project goals and collective conflict management through organizational structure design (Weisenfeld et al., 2010). However, current research relies on traditional employment relationships and overlooks the non-employment challenges ecosystem ventures uniquely face in managing dependent and independent conflicting goals.

Dependent entrepreneurship requires adherence to hub firm goals and value propositions, which may constrain technological development, product design and market deployment, potentially causing “platform traps” (Gawer and Cusumano, 2002). Despite the relative abundance of entrepreneurial resources and opportunities (Adner and Kapoor, 2010; Swanson, 2024), ecosystem entrepreneurs inevitably face seeking independent growth while mitigating negative hub firm impacts, without relying on the hub firm’s success. The capability of alliance management and relational governance serve as instrumental tools for enhancing the success rate of new ventures (Rabelo Neto et al., 2024). Entrepreneurs, in their pursuit of rapid growth, must possess the capacity for self-regulation (Lange et al., 2023). Although scholars have theoretically expounded the positive role of ecosystem entrepreneurs’ cognitive resource mobilization and self-regulation in managing conflicting goals (Nambisan and Baron, 2013; Annosi et al., 2024), empirical analysis is lacking.

This study focuses on Haier Entrepreneurship Ecosystem, exploring how ecosystem entrepreneurs perform self-regulation by mobilizing cognitive resources to construct capabilities for managing dependent and independent conflicting goals. Guiding research questions include: What triggers the construction of these capabilities? What are the construction processes and mechanisms? And how do entrepreneurs mobilize cognitive resources to self-regulate? By investigating the cognitive processes and logic underlying the rational management behavior of ecosystem entrepreneurs, this study aims to supplement the causal research in the “cognition-behavior” empirical framework within the field of entrepreneurship research and provide a basis for the assessment and learning of ecosystem entrepreneurs’ cognitive resources and capabilities.

## 2 Literature review and research framework

### 2.1 Conflict goals management within entrepreneurship ecosystems

When individuals face resource constraints or incompatible strategies for achieving goals, they are unable to pursue multiple objectives simultaneously, leading to the emergence of conflicting goals (Segerstrom and Solberg, 2006). To manage these conflicts effectively, individuals must continuously navigate between distinct, separate goals, a process that requires both shifting and transitioning strategies to maintain the effectiveness of conflict goals management (Tushman and Romanelli, 1985). In the long-term confrontation with conflict goals, there is also a need to seek synergistic management (Jarzabkowski and Sillince, 2007). The concurrent application of these two strategies constitutes the capabilities of conflict goals management.

In the context of entrepreneurship ecosystems, the hub firm and new ventures are interconnected through innovation projects, working collaboratively towards operational sub-goals to achieve the overarching project objectives. In project-based organizations, conflicts arise between project management logic and asset management logic (Sloot et al., 2024). The management of conflicts among these operational sub-goals is pivotal not only to the project’s success but also to the development of the relationship between the hub firm and new ventures, as well as the evolution of the ecosystem itself. To align new ventures with technical specifications and standards, and to govern their key design decisions behaviorally, the hub firm often bolsters its conflict goals management capabilities through ecosystem organization design and platform leadership (Napier et al., 2024).

The dynamics between the hub firm and new ventures within the ecosystem transcend the traditional employment relationship hypothesis posited by conventional organization theory, evolving into diverse and multi-level cooperative relationships (Wenyu et al., 2018). Despite their close association, these entities remain independent, with distinct interests and developmental trajectories. This independence can lead to inconsistencies in the definition and timing requirements of similar goals. The symbiotic relationship between competition and cooperation may trigger conflicting goals (Chatterjee et al., 2023; Zhang et al., 2023), necessitating effective management to maintain equilibrium among the hub firm and new ventures (Batool et al., 2023; Xie et al., 2023). The conflict goals management capabilities of ecosystem entrepreneurs are crucial for identifying opportunities for innovation and growth both within and outside the ecosystem, ultimately facilitating entrepreneurial success (Nambisan and Baron, 2013).

Building upon the existing literature that predominantly examines the hub firm’s perspective, it becomes evident that there is a significant gap in understanding the intricate dynamics from the vantage point of new ventures (Muegge, 2013). While the effectiveness of traditional organizational design methods in managing conflicts is acknowledged, the specific mechanisms by which individual entrepreneurs’ cognition and self-regulation contribute to these capabilities remain underexplored. This gap is particularly pertinent given the complex interplay between competition and cooperation that characterizes entrepreneurial ecosystems. To advance the field, future research should focus on uncovering the micro-foundations of conflict management within new ventures, the role of individual agency, and the interplay between organizational design and entrepreneurial cognition. Such insights are pivotal for enhancing the ecosystem’s adaptability and for equipping new ventures with the tools to navigate goal conflicts more effectively.

### 2.2 Trigger factors and conflict recognition

Conflict goals are not always explicit; they can also exist in a latent state. The recognition of conflict goals’ state is influenced by the presence of certain trigger factors within the objective environment that affect the antagonism and interrelatedness between conflict goals, as well as by the individual’s subjective judgment regarding such antagonism and interrelatedness (Smith and Lewis, 2011). Scholars have emphasized the “driver-process-result” perspective, highlighting how external pressures trigger individual self-regulation, stimulating cognitive renewal to filter situational information in complex and changing environments, such as potential opportunities from policy changes, thereby advancing co-creation of value (Jia et al., 2023).

Researchers have identified various trigger factors that precipitate the explicit manifestation of conflict goals. These include environmental diversification, environmental variability, and resource scarcity. Environmental diversification escalates uncertainty and renders the competing goals and their inconsistent processes more apparent (Denis et al., 2007). Environmental variability introduces new opportunities for managers to address both long-term and short-term conflict goals, as well as the conflict goals management arising from concurrent participation in differing roles (Huy, 2002). Meanwhile, the allocation of scarce resources exacerbates the conflict relationship between mutually opposing and interdependent alternatives (Smith and Tushman, 2005). When combined, these trigger factors exert a more potent influence in the context of globalization and technological innovation.

Within the entrepreneurship ecosystem, environmental changes triggered by these factors can partially or radically alter the power dynamics between the hub firm and new ventures (Yao et al., 2022), thereby stimulating entrepreneurs’ awareness of the antagonism and interrelatedness of conflict goals. This, in turn, impacts their management behavior and capability development (Mitchell et al., 2007). Strategic misalignments, opposing views on operational processes, lack of trust, and asymmetric interdependencies can all serve as specific triggers for conflicts (Xu et al., 2023).

Despite the foundational insights provided by the existing body of work on conflict goals and their triggers within entrepreneurial ecosystems, there is a notable absence of research that delves into the micro-level cognitive mechanisms underlying entrepreneurs’ recognition and management of these goals. The literature has established the significance of environmental factors such as diversification, variability, and resource scarcity in precipitating the explicit recognition of conflict goals. However, it falls short in addressing how these factors interplay to influence entrepreneurial cognition and the subsequent strategic responses. A critical gap exists in understanding the adaptive strategies that entrepreneurs employ in response to environmental triggers and how these strategies impact the development of conflict management capabilities. The current research also lacks a comprehensive examination of the long-term implications of conflict goal management on the entrepreneurial ecosystem’s evolution and stability. Future research should aim to bridge these gaps by focusing on the cognitive and behavioral dimensions of entrepreneurs’ interactions with trigger factors. This includes a detailed exploration of the decision-making processes that lead to the recognition of conflict goals and the development of targeted management practices. Additionally, longitudinal studies that trace the trajectory of conflict goal management and its impact on ecosystem dynamics would significantly enhance the field’s understanding of these complex phenomena.

### 2.3 Self-regulation and conflict goals management capability

The cognitive processes and efforts of entrepreneurs significantly influence entrepreneurial behavior, as highlighted in the literature (Baron and Henry, 2010). For example, some scholars emphasize the importance of reflection for entrepreneurs when confronting the inherent uncertainties in the entrepreneurial process (Muñoz and Dimov, 2023). Some scholars have proposed the concept of individual psychological ownership (PO), which refers to how individuals develop ownership of a certain goal through control, intimate understanding, and self engagement (Kim et al., 2024). These can provide insights for entrepreneurs on how to manage conflict goals through cognitive effort. Against the backdrop of globalization and technological advancements, ecosystem entrepreneurs enhance organizational adaptability and dynamic capabilities through cognitive efforts and conflict goals management capabilities (Feng et al., 2022; Sjödin et al., 2024).

The engagement of effort and focus on competing goals within the realm of conflict goals management is conceptualized as “self-regulation” (Schmidt et al., 2009), which involves how entrepreneurs control and integrate trigger factors into their self-identity (Schwarte et al., 2023). It is a process characterized by the continuous overcoming of resistance to develop capabilities (Grund and Fries, 2018; Zhang et al., 2023). Key components of self-regulation include self-control, grit, and metacognition, each playing a distinct role in the entrepreneurial journey.

Self-control is pivotal in the goal pursuit process (Fujita, 2011), ensuring the accuracy of goal direction, facilitating timely adjustments between conflict goals, and encompassing the regulation of thoughts, feelings, and behaviors (Tushman and Romanelli, 1985). In the context of entrepreneurship ecosystems, the self-control of entrepreneurs positively influences the management of conflicting performance goals, technological development goals, and relational goals between the hub firm and new ventures (Nambisan and Baron, 2013).

Grit, as defined by perseverance in pursuing long-term goals, is manifested through sustained effortful behavior despite challenges and temptations, alongside a persistent interest in goals (Duckworth and Quinn, 2009). Differing from self-control, which primarily addresses immediate behavior, grit pertains to the control process over an extended period (Eskreis-Winkler et al., 2014). The grit of ecosystem entrepreneurs positively impacts the management of conflicting technological development goals and relational goals between the hub firm and new ventures (Nambisan and Baron, 2013).

Metacognition involves the mental structures, knowledge, events, and processes that govern, modify, and interpret thought processes (Wells and Cartwright-Hatton, 2004). As a higher-order cognitive function, metacognition facilitates the self-regulation process by enhancing cognitive adaptation, enabling information interaction with the environment, and responding to feedback (Haynie et al., 2010). The metacognitive abilities of ecosystem entrepreneurs positively influence the management of conflicting technological development goals between the hub firm and new ventures (Nambisan and Baron, 2013).

While the literature has provided a robust framework for understanding the role of cognitive processes in entrepreneurial behavior, a significant gap persists in comprehending the synergistic effects of self-control, grit, and metacognition on conflict goals management. The current research has predominantly focused on the individual impact of these cognitive dimensions, often in isolation, which may not fully encapsulate the multifaceted nature of entrepreneurial cognition in ecosystems (O'Shea et al., 2017). The mechanisms that underlie the integration of these cognitive resources into a cohesive self-regulation strategy, particularly within the dynamic entrepreneurial context, are not well articulated. Future research should prioritize the development of integrative models that can account for the complex interplay among cognitive resources. This includes examining how entrepreneurs leverage these resources in concert to navigate conflict goals and the formation of management capabilities. Empirical studies that employ a longitudinal approach to observe the evolution of cognitive processes and their strategic application in real-time settings will be instrumental in filling these research voids.

**Figure 1:**
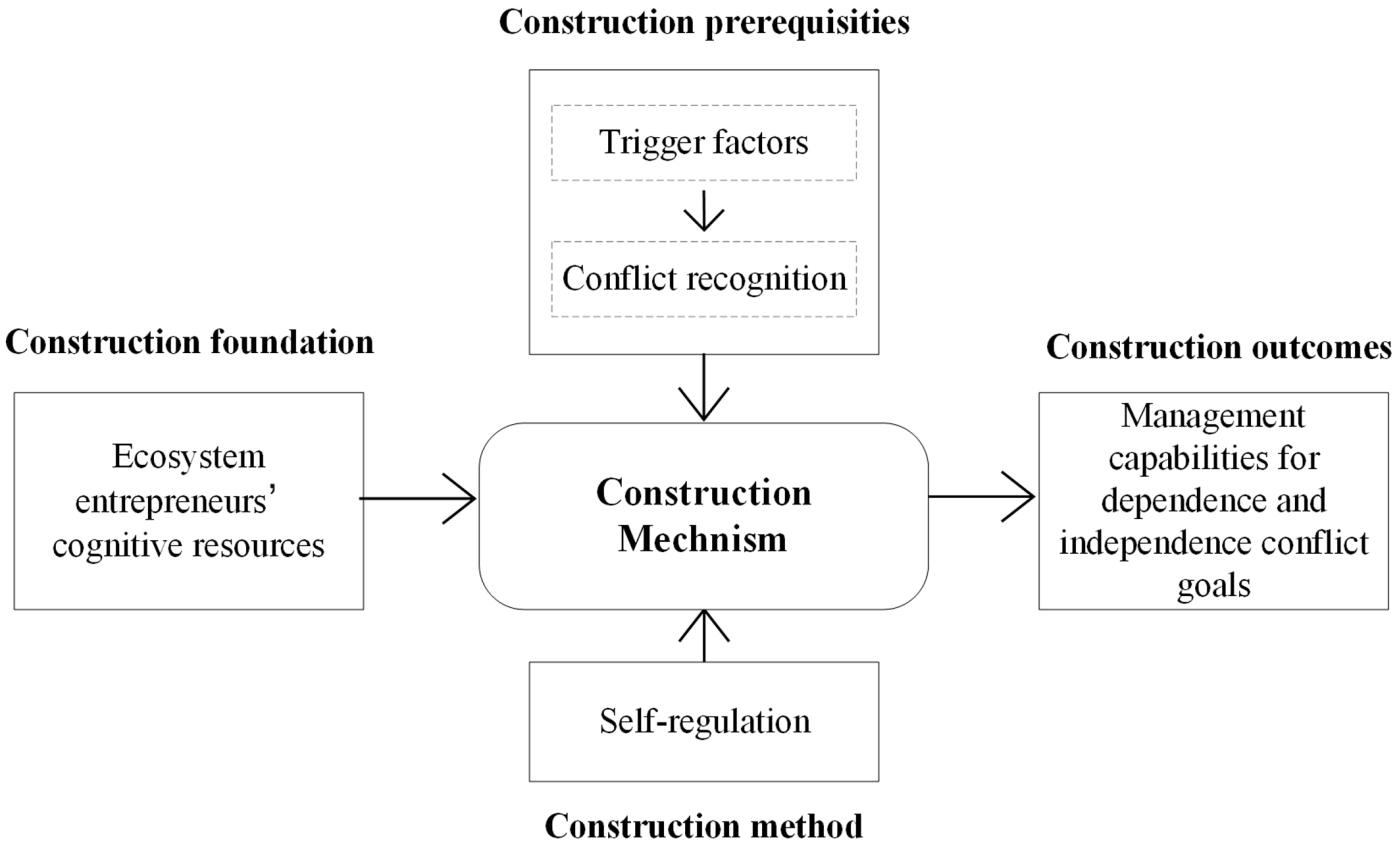
Research framework.

In summary, existing research has confirmed the positive impact of ecosystem entrepreneurs’ self-regulation processes on the development of conflict goals management capabilities. However, there is still a lack of comprehensive descriptions regarding the construction mechanisms. This paper aims to delve into the establishment of ecosystem entrepreneurs’ management capabilities for both dependent and independent conflict goals. It does so by considering cognitive resources as the foundational elements, self-regulation as the construction method, and the entrepreneurs’ conflict recognition triggered by external factors as the basis for the construction process. The research framework is illustrated in Figure 1.

## 3 Materials and methods

### 3.1 Research method

This paper adopts an exploratory single-case study approach to investigate the mechanisms by which ecosystem entrepreneurs develop conflict goals management capabilities through self-regulation. The choice of this approach is driven by two main reasons. Firstly, as the cumulative impact of various cognitive elements of entrepreneurs on the construction of conflict goals management capabilities is still relatively nascent in academic research, the case study method is well-suited for addressing research questions such as “how” and “why” (Jiye, 2020). Secondly, a single-case study is particularly effective in refining and theoretically inducing phenomena within specific contexts (Gioia et al., 2013), allowing for a more in-depth analysis of the core essence of complex issues within the entrepreneurship ecosystem context.

### 3.2 Case selection

Adhering to the principles of theoretical sampling, typicality, and heuristic value, this paper selects Haier Group (hereafter referred to as “Haier”) as the case sample, focusing on entrepreneurs within the Haier entrepreneurship ecosystem for the case analysis.

Founded in 1984, Haier is a leading global provider of better life and digital transformation solutions. Based on the purpose of “More Creation, More Possibilities.” Haier is committed to co-create infinite possibilities for a better life with users, and to co-create infinite possibilities for industrial development with the ecosystem partners. Being an iconic company in the real economy, Haier has always been user centered, adhered to original technology and built a landscape of two pillars, Smart Living and Industrial Internet. Haier has built 10 R&D centers, 71 research institutes, 35 industrial parks, 143 manufacturing centers, and a sales network of 230,000 nodes globally (For more information, please refer to the website https://www.haier.com).

Firstly, the principle of theoretical sampling necessitates that the case subject adequately reflects the main relationships between the constructs involved in the research question. As of February 2024, the Haier entrepreneurship ecosystem has incubated 7 unicorn companies, 107 gazelle companies, and 175 specialized and sophisticated “small giant” enterprises, in addition to more than 360 key accelerated enterprises and over 5,200 entrepreneurial projects. Entrepreneurs rely on Haier for entrepreneurial resources and opportunities, and at the appropriate time, new ventures become independent to pursue greater development opportunities. This dynamic reflects the practical challenges faced by entrepreneurs in the Haier ecosystem when managing capabilities for dependent and independent conflict goals.

Secondly, the case’s typicality and uniqueness are paramount. Haier is the only IoT Ecosystem Brand in the world to have been ranked in the Kantar BrandZ Top 100 Most Valuable Global Brands for 5 consecutive years. Additionally, Haier has maintained the top position in Euromonitor’s Global Major Appliances Brand for 15 consecutive years. As an industry leader, Haier aligns with the requirements for a typical case study.

Thirdly, the heuristic value of the case study is significant. The formation of the Haier entrepreneurship ecosystem has undergone various modes and forms, including the stimulation of autonomy and innovative capacity of grassroots employees in traditional manufacturing, the internal drive potential mining under the interaction between the entrepreneurial platform and makers during the transformation period, and the chain-cluster contracts ignition under the IoT community ecology through iterative interactions between the entrepreneurial platform, the new ventures, and users. Its exploratory experience and operational methods can offer valuable insights into the development of conflict goals management capabilities for ecosystem entrepreneurs.

### 3.3 Data collection

Between July 2019 and July 2023, the research team utilized various methods for data collection, including semi-structured interviews, archival data, field observations, and informal communication (refer to Table 1). Employing a triangulation approach with multi-source data helped mitigate retrospective bias and ensured the depth and credibility of the data. Semi-structured interviews served as the primary data source for this study, while field observations and informal communication further enriched the research team’s understanding of the Haier ecosystem and its entrepreneurs.

**Table 1:** Case data source and core contents.

Data source		Statistical information		Data description
Semi-structured interviews	Ecosystem entrepreneur	12 person-times	23 h	12 entrepreneurs in 8 ventures
	Hub firm manager	3 person-times	4 h	3 department managers of Haier Group
Archival data	Academic report	22 copies	198 pages	Academic reports on the new ventures in Haier Group in the past 3 years
	Related book	4 copies	1,116 pages	Related books such as “Black Sea Strategy: How Haier Builds a Platform Ecosystem”
	Media report	85 copies	267 pages	Mainstream media reports, WeChat official account push, management interviews, etc. in recent 3 years
	Internal documents	93 copies	744 pages	Haier People’s internal publication, business presentation PPT documents, etc.
On site observation	Field note	5 meetings	28 pages	3 Haier RenDanHeYi conferences and 2 seminars on “Haier Ecosystem Rainforest Plan”
	Field note	6 visits	38 pages	Two visits to Haier headquarters and four visits to the new ventures
	Field note	3 store surveys	15 pages	Internet of Things Store 001 and 3 other stores
Informal communication	Memory note	8 person-times	17 pages	8 familiar internal employees of Haier

### 3.4 Data analysis

A three-level coding method was applied for the grounded analysis of the data. Initially, two researchers independently reviewed the data, coding it sentence by sentence, and then compared their results. Subsequently, other team members reviewed and refined the codes to form preliminary first-order concepts. Following this, researchers explored the organic connections between the first-order concepts to develop abstracted, theoretical second-order themes. Finally, researchers independently integrated these second-order themes into aggregate constructs, with all members engaging in collective discussions. This iterative process involved multiple rounds of revision. Throughout the analysis, coded data were consistently reviewed by interviewees for validation, supplementation, or exclusion to rectify any data biases. Moreover, theoretical experts were consulted to verify the findings and contribute to discussions, ensuring the accuracy and reliability of the case study results.

## 4 Case analysis and results

This paper conducts a case analysis of entrepreneurs within Haier to synthesize the trigger factors, conflict recognition, and construction mechanisms of ecosystem entrepreneurs’ management capabilities for dependent and independent conflict goals. The details are elaborated below.

### 4.1 Trigger factors to develop management capabilities of conflict goals

Strategic corresponding and mechanism adapting are identified as the two trigger factors for ecosystem entrepreneurs to develop management capabilities of conflict goals, with core coding and key evidence presented in Table 2.

**Table 2:** Core coding and key evidence of trigger factors to develop management capabilities of conflict goals.

Construct	Second order theme	First order concept	Key evidence
Strategic corresponding	Adjustment and change of ecosystem strategy	Periodic changes in ecosystem strategy	“At different stages, the hub firm will have different strategies that will be adjusted. The new ventures need to match their strategies in order to develop.” (P01)
		The hub firm deploys the new ventures according to strategy	“The hub firm forcefully separates the new ventures that are unrelated to its main business, leaving behind the group’s desire to make this area a subsidiary business of its own.” (E05)
	The strong influence of ecosystem strategy	Resource acquisition relies on ecosystem strategy	“But there will be a focus on the service, and when my (the hub firm’s) resources are insufficient, I (the hub firm) will first satisfy my balance.” (P02)
		Business that does not align with the ecosystem strategy cannot be carried out	“Because the industry we are in is not Haier’s main business, many things were not approved within Haier at that time, so this industry could not be developed at that time. If we violated the process within Haier and were eventually punished.” (E03)
Mechanism adapting	The driving mechanism changes and develops	Mechanism changes enhance the new ventures’ vitality	“The new ventures’ vitality was insufficient. Later, it was mainly through changes in the mechanism. Haier’s previous process was very complicated. It was difficult to push things forward, especially when they were unconventional.” (E04)
		Ecosystem launch new driving mechanisms	“For example, the ecosystem is now pushing chain-group cooperation, which is a new driving mechanism.” (P01)
	The driving mechanism has strong binding force	The driving mechanism has a whole constraint on the new ventures	“The ecosystem saw that there was a problem with one of the new ventures. This kind of exposure might be an overall problem. If they found such a hidden problem, they would adjust the mechanism. However, the ecosystem would not study every new venture one by one.” (E05)
		The new ventures competition and elimination through the driving mechanism	“The original efficiency is not high, now changed the mechanism, directly on the platform to issue orders, you all come to snatch orders, competition and elimination. If the entrepreneur has not been able to lead the team to a virtuous circle, the next time to snatch orders, he is likely to be PK down.” (P03)

The hub firm formulates a development strategy and implements ecosystem operations and business to the new ventures. This strategy determines the acquisition of resources and services for the new ventures, as well as their business scope, exerting a significant influence on their development. The new ventures are obligated to unconditionally fulfill the ecosystem’s strategic needs set by the hub firm. If the new ventures are related to the core business of the hub firm, they will continue to be incubated. For those outside the ecosystem, the hub firm may strategically retract them, thereby triggering and explicitly manifesting the conflict between dependence and independence goals. Conversely, if the new ventures are misaligned with the ecosystem strategy, such as being irrelevant to the core business or inconsistent with the mainstream process, they may experience a reduction in resource supply to decrease their dependence or proactively seek independence to increase their autonomy, thus also triggering and making explicit the conflict between dependence and independence goals.

Additionally, the hub firm establishes driving mechanisms to ensure the efficient and orderly operation of the vast ecosystem. The new ventures achieve development and growth through mechanism adaptation. If the development stage of these entities aligns with the ecosystem driving mechanisms, they may actively choose to remain attached, thus entering a virtuous cycle of rapid development, or be attracted by the mechanisms to move from outside to inside the ecosystem, thereby triggering and making explicit the conflict between dependence and independence goals. If the development stage of the new ventures does not align with the driving mechanisms, one scenario is that the new ventures rapidly mature and grow through leapfrog development, gaining more autonomy and seeking independence outside the ecosystem. Another scenario is that if the new ventures are underperforming, the hub firm may exert pressure to push them out of the ecosystem, forcing independence and thus also triggering and making explicit the conflict between dependence and independence goals.

### 4.2 State recognition to develop management capabilities of conflict goals

Ecosystem entrepreneurs perceive the conflict between dependence and independence goals through strategic corresponding and mechanism adapting as a state of equilibrium, while the conflict arising from strategic misalignment or mechanism incompatibility is perceived as a state of disequilibrium. The core coding and key evidence for this perception are presented in Table 3.

**Table 3:** Core coding and key evidence of state recognition to develop management capabilities of conflict goals.

Construct	Second order theme	First order concept	Key evidence
Equilibrium	The natural existence of dependent and independent goals	Want to be independent as well as dependents	“(The new venture) is willing to (stay in Haier) when it has resources, not when it does not want to be managed by you (Haier), and is not willing to attend meetings every day.” (P03)
		Different goals have different requirement	“The new venture will have a priority to meet such goals as those from Haire, chain group, and its own, because these three goals will have different requirements.” (P01)
	The implicit opposition between dependence and independent goals	There is not much concern about dependence or independence goals	“I do not think he (entrepreneur) will deliberately adjust his attitude. Is he dependent on Haire or independent? I do not think he will think too much about it.” (E05)
		Both goals can be achieved at the same time.	“We have achieved the goals of the chain group and our own at the same time. Usually they do not conflict. It does not mean that I can not accomplish this and that.” (P02)
Disequilibrium	Break the equilibrium actively.	Want to get out of Haire	“Our bank account is connected back to Haire’s system. We have to go through Haire’s system to pay and collect money. If we have to review it, it will affect the flexibility of our business. We want the process to be fast and efficient. You are bound by this thing, you can not run fast, so want to get out.” (E05)
		Want to go into Haire from outside	“Doodling is also one of the ventures, outside entrepreneurs come in, according to ecosystem direction of screening in.” (E02)
	Break the equilibrium passively	There are ventures that must be pushed out of the ecosystem	“When Haire was miniaturized, every platform or industry had to have a few hatchlings. I remember it was like this at that time. It was hard to push at first, and many did not want to leave.” (P03)
		There are ventures that must be take back to strengthen management	“The home appliance market has not been good for the past 2 years. The growth rate is very slow, and the industry is declining. The whole industry of the group is now shrinking, so we will take back these ventures to strengthen management.” (E05)

In a state of equilibrium, the dependence and independence goals do not inherently oppose each other; instead, they coexist and develop in a stable confrontation. Although the natural existence of conflicting goals of dependence and independence presents various sub-goal requirements to the new ventures, ecosystem entrepreneurs do not recognize the opposition of conflicting goals at this time. However, when there is a strategic misalignment or mechanism incompatibility, the state of equilibrium is disrupted. Ecosystem entrepreneurs will lead the new ventures to actively exit or be passively pushed out of the ecosystem, or entrepreneurs outside the ecosystem will lead the new ventures to actively enter or be forcibly pulled into the ecosystem.

**Figure 2:**
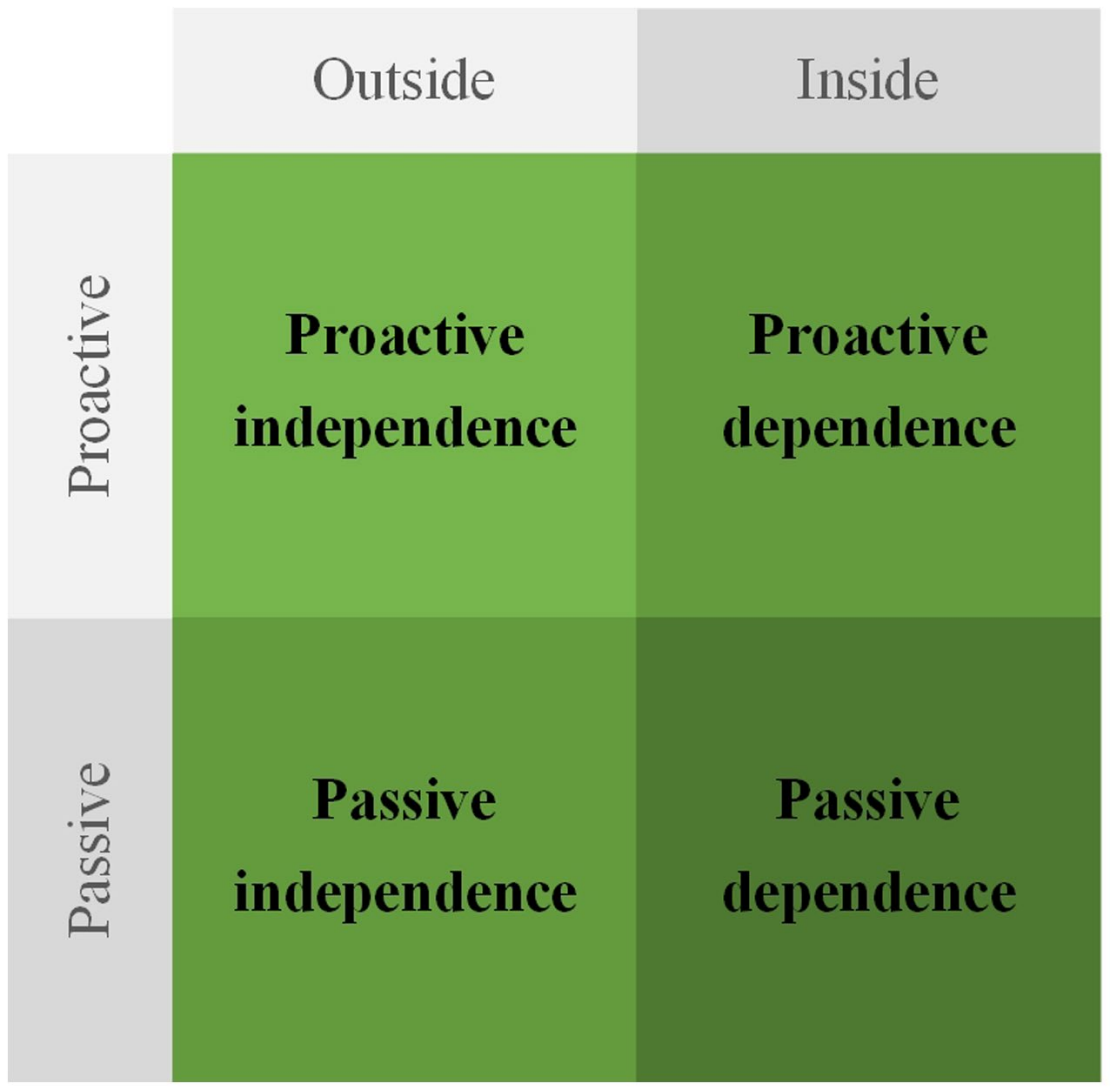
Four manifestations of the disequilibrium state of conflict goals.

Depending on entrepreneurs’ intentions, performance, and the resulting relationship with the ecosystem, the state of disequilibrium mainly manifests in four scenarios: proactive independence, passive independence, proactive dependence, and passive dependence (see Figure 2). Proactive independence occurs when ecosystem entrepreneurs rely on their own strength to increase autonomy and reduce dependency, actively distancing themselves from the hub firm and the ecosystem. Passive independence is when the hub firm changes the resource supply or conditional constraints for the new ventures, forcing the entrepreneurs to increase autonomy and reduce dependency, and passively distancing themselves from the hub firm and the ecosystem. Both proactive and passive independence are characterized by a trend of exiting the ecosystem. Proactive dependence is when entrepreneurs rely on their own strength to increase dependency and reduce autonomy, actively drawing themselves closer to the hub firm and the ecosystem. Passive dependence occurs when the hub firm changes the resource supply or conditional constraints for the new ventures, forcing the entrepreneurs to increase dependency and reduce autonomy, and passively drawing themselves closer to the hub firm and the ecosystem. Both proactive and passive dependence are characterized by a trend of entering the ecosystem.

### 4.3 Construction mechanisms to develop management capabilities of conflict goals

The construction mechanisms for developing management capabilities of conflicting goals among ecosystem entrepreneurs involve two distinct approaches: the decoupling mechanism based on antagonistic goals recognition and the coupling mechanism based on synergistic goals recognition. The core coding and key evidence for these mechanisms are detailed in Table 4.

**Table 4:** Core coding and key evidence of construction mechanisms to develop management capabilities of conflict goals.

Construct	Second order theme	First order concept	Key evidence
Decoupling	Opposite consciousness	There are advantages and disadvantages to mechanism control	“When the group is controlling you, it is actually using Haier’s years of management experience and problems that may arise in the project to control you in advance by considering the risk points to be avoided. In fact, this is a kind of growth and help for ventures, I think so, of course, more than that is not necessarily.” (E05)
		Entrepreneurs worry about strategic constraints	“My biggest concern right now is that it can be very troublesome if it interferes with the larger direction of my company.” (E01)
	Dissociative behavior	Entrepreneurs insists on a clear direction different from Haier’s	“Haier’s request is useless, I can only say that I can afford to try. This is something that has to be done. I’ve always agreed to do it, but not at this stage. This is my direction, I am very clear this direction.” (E01)
		Entrepreneurs take the initiative to enter new industries	“A lot of things were not approved within Haier at that time, and once you did it, you broke the process within Haier. So in this case, let the team have greater autonomy to enter this space (new industry). (E04)
Coupling	Holistic consciousness	The hub firm and new ventures have the same vision	“We have a big strategy for the new ventures to focus on where we are going. In fact, our vision is certain, is a good family solution. But in different stages, we will have different strategies to help you achieve.” (P01)
		The new ventures are in line with the hub firm’s goals	“I agree with Haier’s vision, but the method and the path are different, the steps are different, the time is not the same.” (E01)
	Cooperative behavior	Contradictory goals can be satisfied by internal logic	“All things, all goals I want to meet, is seemingly contradictory. In fact, it is internally logical.” (E03)
		The goals can be integrated into an order	“The entrepreneur turns the group’s strategy into a goal, and then his goal, which is a collection of goals. Integration of the whole order (Dan of RenDanHeYi), is an order set. There may be some synergy in this.” (P01)

The decoupling mechanism, activated when the conflict goals exist in a state of equilibrium, involves ecosystem entrepreneurs engaging in self-regulation to enhance the recognition of the antithetical nature of conflict goals and to implement segregated management practices. This approach ensures the preservation of independence within dependence and dependence under independence as both sets of goals evolve concurrently. In an equilibrium state, despite the absence of overt antagonism between the dependence and independence goals, entrepreneurs mobilize cognitive resources to establish a conscious awareness of the dichotomy between these interdependent goals. Specifically, entrepreneurs maintain a vigilant and discerning perspective regarding the constraints and strategic limitations imposed by the hub firm. Additionally, these entrepreneurs venture beyond the directives of the hub firm, proactively generating a broader array of developmental opportunities and prospects.

Conversely, the coupling mechanism comes into effect when the conflict goals are identified in a state of disequilibrium. Ecosystem entrepreneurs apply self-regulation to foster a holistic understanding of conflict goals and to coordinate management efforts. This strategy intensifies the synergy between dependence and independence goals during their misaligned growth, thereby facilitating better adaptation to and control over the imbalanced state. In conditions of disequilibrium, the inherent opposition between dependence and independence goals becomes more pronounced. Entrepreneurs exert cognitive efforts to reconstitute a comprehensive consciousness of the interconnected dependence and independence goals, namely by reaffirming and emphasizing a shared vision and congruent objectives with the hub firm. Moreover, entrepreneurs seek to find logical coherence between conflict goals, striving for synergy and fulfillment through a focus on “Dan” (one word from “RenDanHeYi,” meaning order within the group).

### 4.4 Construction outcomes to develop management capabilities of conflict goals

The outcomes of the construction of management capabilities for conflict goals among ecosystem entrepreneurs encompass both segregative and synergistic management capabilities, with core coding and key evidence presented in Table 5.

**Table 5:** Core coding and key evidence of construction outcomes to develop management capabilities of conflict goals.

Construct	Second order theme	First order concept	Key evidence
Management capabilities of conflict goals	Segregative management capabilities	In the equilibrium state, the original business with strong dependence gradually increases independence, and the team autonomy is enhanced in the progressive change	“It used to be managed by Haier, but considering that our industry is not the main business of Haier, it can not be done within Haier. So we want to give the team more autonomy. This change is the biggest.” (E05)
		In the equilibrium state, the original independent business gradually increases its dependence and eventually becomes new ventures	“I was in charge of the global business of Haier BJB. In fact, at that time, I thought of BJB as the latest terminal of the Internet. How should we practice this management theory, or even practice the organizational form, I did not know how to do it at first. But this thing is starting to happen, ... ... We start researching, exploring, collecting users’ needs and complaints, combining what we have with what we can integrate. After about 7 or 8 months of preparation, the first batch of products came to market. This is why we did, how we did, and how we got it in the first place. After this matter has gone forward, it was actually not a new venture.” (E01)
	Synergistic management capabilities	In the disequilibrium state, the passive independent business seeks the greater dependence, and finally realizes the self-operation	“At that time, the group did not want us anymore. If we could not find buyers, we would have to close down. After that, our team found a lot of small investors and found a small fund management company. We pooled the money and gave it to Haier. Because at that time, the company was supported by Haier real estate, and the profits from the orders were about 30 million a year. From 2014 to the beginning of 2016, we made the company profitable and self-sustaining.” (E04)
		In the disequilibrium state, the passive dependence business seeks greater independence, and ultimately achieve a win-win relationship between the hub firm and new ventures	“As we got bigger, Haier had some ideas, and we gave the company back. In fact, since its founding, LX has not been invested much money by Haier. But Haier has recovered 35 million in cash and nearly $400 million worth of shares. We can use its resources, such as after-sales, logistics, supply chain, goodwill. But we do not use Haier’s product channels and brands.” (E04)

In a state of equilibrium, entrepreneurs construct segregative management capabilities for conflict goals of dependence and independence by contemplating and enacting independent goals, thereby acquiring greater autonomy and independence for their ventures. Entrepreneurs mobilize cognitive resources and utilize self-regulation to incrementally infuse independence into operations that were previously highly dependent, thereby enhancing team autonomy through progressive transformation. Conversely, they may gradually introduce dependence into operations that were originally more independent, ultimately becoming closely connected with the hub firm. Through this behavioral process, the segregative management capabilities gradually take shape.

In a state of disequilibrium, entrepreneurs build synergistic management capabilities for conflict goals of dependence and independence by considering and implementing collaborative goals, thus gaining greater initiative and dependence for their enterprises. Entrepreneurs leverage cognitive resources and self-regulation to seek increased dependence for operations that are passively independent, aiming for self-sustaining functionality. Similarly, they strive for greater independence in operations that are passively dependent, ultimately achieving a win-win situation with the hub firm. Throughout this behavioral process, the synergistic management capabilities are progressively developed.

### 4.5 Ecosystem entrepreneurs’ self-regulation process in the construction mechanism

Ecosystem entrepreneurs’ self-regulation process in the construction mechanism is a crucial aspect of their behavioral projections. The unseen cognitive processes play a key role in shaping their behavior (Bird et al., 2012). This study aims to further explore the role of ecosystem entrepreneurs’ cognitive resources and their self-regulation process based on theoretical research.

During the construction process of the decoupling mechanism, which is based on antagonistic goals recognition, ecosystem entrepreneurs undergo a cognitive deepening from synergistic goals recognition to antagonistic goals recognition. Triggered by external environmental factors, this process enters the internal cognitive system of the entrepreneur, sparking a cognitive response that identifies the opposition of conflict goals in a state of equilibrium. The process involves the deconstruction of dependent and independent goals, clarifying the strong synergy and weak opposition of conflict goals in equilibrium. This is followed by mobilizing cognitive resources to reinforce the oppositional relationship, ultimately forming antagonistic goals recognition.

In the construction process of the coupling mechanism, based on synergistic goals recognition, ecosystem entrepreneurs complete a cognitive deepening from antagonistic goals recognition to synergistic goals recognition. Triggered by external environmental factors, this process enters the internal cognitive system of the entrepreneur, sparking a cognitive response that identifies the synergy of conflict goals in a state of disequilibrium. The process involves the deconstruction of dependent and independent goals, clarifying the weak synergy and strong opposition of conflict goals in disequilibrium. This is followed by mobilizing cognitive resources to reinforce the synergistic relationship, ultimately forming synergistic goals recognition.

Although the content of the two mechanisms differs, the underlying construction follows the same logic of self-regulation, encompassing components such as environmental interaction perception, conflict goals analysis, and goals relationship definition. The specific contents include perceiving the equilibrium and disequilibrium states of conflict goals of dependence and independence, mobilizing cognitive resources to form a self-regulation process, forming an alternative cognition of associated goals based on self-regulation, and applying alternative cognition to the management behavior of conflict goals.

### 4.6 Mobilization and function of cognitive resources in the self-regulation

**Figure 3:**
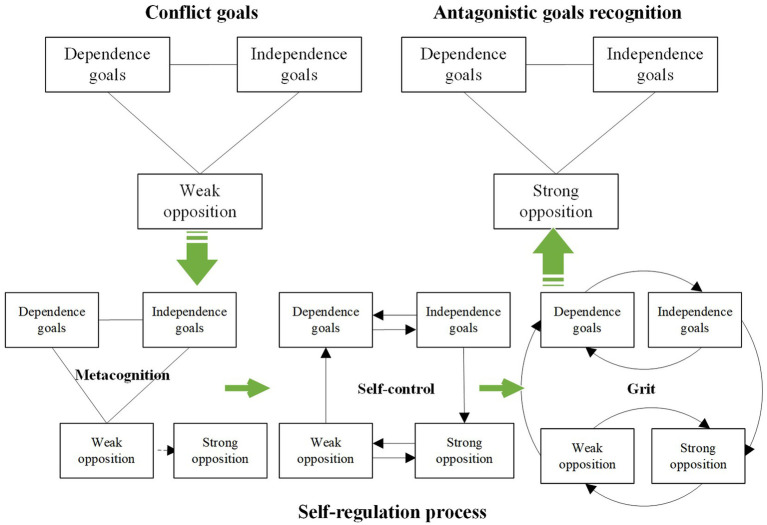
The self-regulation process of antagonistic goals recognition.

**Figure 4:**
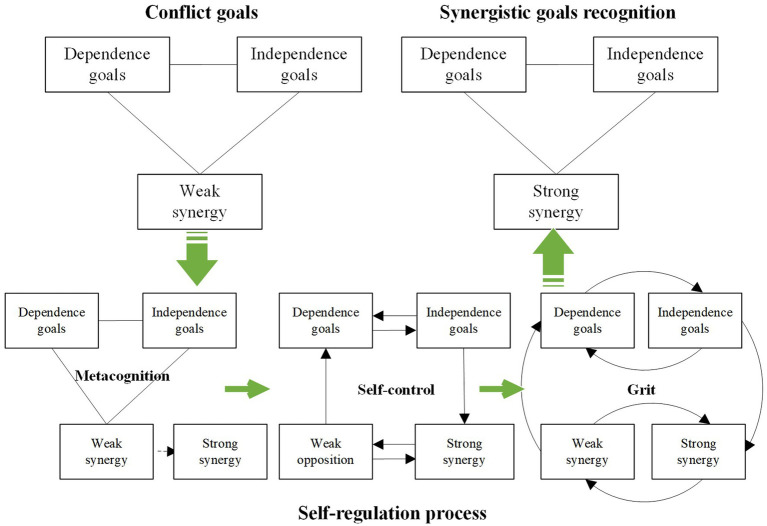
The self-regulation process of synergistic goals recognition.

Different cognitive resources yield distinct outcomes in the self-regulation process. Metacognition aids in environmental recognition and analysis, as well as the formulation and assessment of alternative solutions; self-control underscores the directional management of individual impulses, thoughts, attention, and behaviors, while grit signifies sustained interest and continuous action. In conjunction with the self-regulation process for constructing conflict goals management capabilities, ecosystem entrepreneurs must mobilize cognitive resources to interact with trigger factors, engage in transformative thinking and conduct iterative analysis of deconstructed dimensional goals. They also need to develop and evaluate alternative plans. Table 6 summarizes the mobilization and function of cognitive resources in self-regulation during the construction process of conflict goals management capabilities by ecosystem entrepreneurs. The self-regulation processes for antagonistic goals recognition and synergistic goals recognition are detailed in Figures 3, 4, respectively.

**Table 6:** The mobilization and function of cognitive resources in self-regulation to develop management capabilities of conflict goals.

Function of cognitive resources		Antagonistic goals recognition	Synergistic goals recognition
Metacognition	Environment identification and interaction	Identification of weak opposition between dependent and independent conflict goals in equilibrium state.	Identification of weak synergy between dependent and independent conflict goals in disequilibrium state.
	Develop and evaluate alternatives	Break through the existing cognition of relevance goals and produce the substitute cognition of strong opposition relation.	Break through the existing cognition of the relevant goals and produce the substitute cognition of strong synergy relation.
Self-control	The thinking transformation of the goals content dimension	From thinking about “dependent goals” to thinking about “independent goals”; from thinking about “independent goals” to thinking about “dependent goals.”	From thinking about “dependent goals” to thinking about “independent goals”; from thinking about “independent goals” to thinking about “dependent goals.”
	The thinking transformation of the goals relationship dimension	From thinking about “weak opposition” to thinking about “strong opposition”; from thinking about “strong opposition” to thinking about “weak opposition.”	From thinking about “weak synergy” to thinking about “strong synergy”; from thinking about “strong synergy” to thinking about “weak synergy.”
	The thinking transformation of the goals from content to relationship dimension	From thinking about “weak opposition” to thinking about “dependent goals”; from thinking about “independent goals” to thinking about “strong opposition.”	From thinking about “weak synergy” to thinking about “dependent goals”; from thinking about “independent goals” to thinking about “strong synergy.”
Grit	The continuous behavior and interest of the goals content dimension thinking transformation	Constantly switch between thinking about “dependence goals” and “independence goals” and maintain a sustained interest.	Constantly switch between thinking about “dependence goals” and “independence goals” and maintain a sustained interest.
	The continuous behavior and interest of the goals relationship dimension thinking transformation	Constantly switch between thinking about “weak opposition” and “strong opposition” and maintain a sustained interest.	Constantly switch between thinking about “weak synergy” and “strong synergy” and maintain a sustained interest.
	The continuous behavior and interest of the goals content to relationship dimension thinking transformation	Constantly switch between thinking about “weak opposition” and “dependence goals,” between thinking about “independence goals” and “strong opposition,” and maintain sustained interest.	Constantly switch between thinking about “weak synergy” and “dependence goals,” between thinking about “independence goals” and “strong synergy,” and maintain sustained interest.

**Figure 5:**
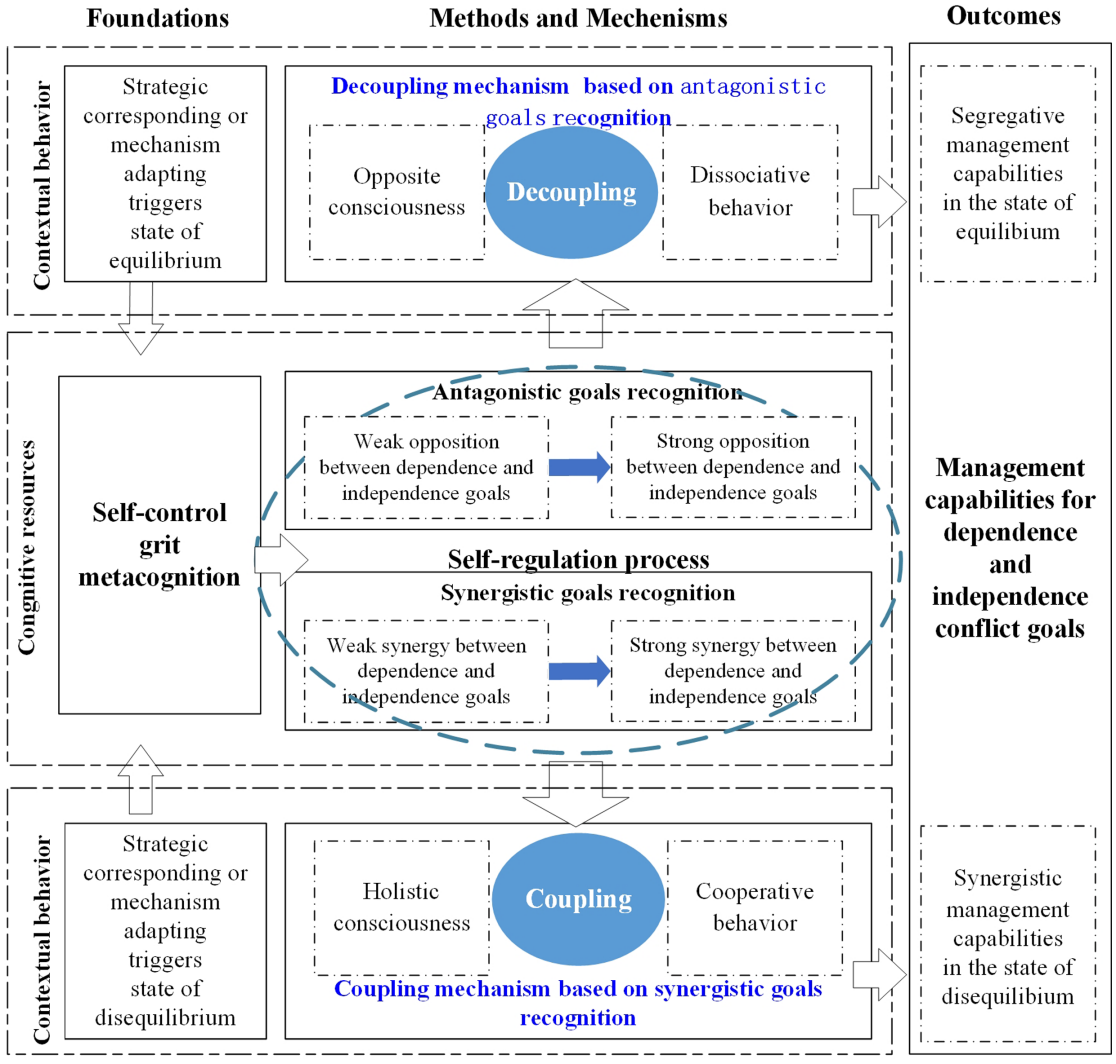
Theoretical model for constructing conflict goals management capabilities based on entrepreneurs’ self-regulation.

Through case analysis, it has been found that cognitive resources such as self-control, grit, and metacognition are foundational for the construction of management capabilities for conflicting goals of dependence and independence. Strategic corresponding and mechanism adapting are the two trigger factors. The decoupling mechanism is based on antagonistic goals recognition, while the coupling mechanism is based on synergistic goals recognition. The outcomes of this process are segregative management capabilities and synergistic management capabilities, which are prerequisites for capabilities construction. Accordingly, this paper proposes a model for constructing conflict goals management capabilities based on entrepreneurs’ self-regulation, as seen in Figure 5.

## 5 Implications and limitations

### 5.1 Research conclusions

The conclusions of this study are as follows: (1) The process of constructing management capabilities for conflict goals of dependence and independence among ecosystem entrepreneurs encompasses multiple levels and dimensions, including conflict cognition, and mechanism design; (2) Trigger factors consist of strategic corresponding and mechanism adapting. Conflict cognition manifests as equilibrium and disequilibrium conflict goals recognition, with two distinct mechanisms being decoupling mechanism based on antagonistic goals recognition and coupling mechanism based on synergistic goals recognition; (3) Cognitive resources such as self-control, grit, and metacognition play different roles in the self-regulation process, exerting a composite impact on the construction of conflict goals management capabilities.

### 5.2 Theoretical significance

A pivotal aspect that distinguishes this study lies in its integrative approach to understanding the complexities of conflict goals management within entrepreneurial ecosystems. The research pioneers a novel framework that encapsulates the multi-tiered process of capability construction, moving beyond a unidimensional view to acknowledge the intricate interplay of conflict cognition and mechanism design. The theoretical contributions of this study are primarily reflected in three aspects:

Firstly, the research extends the cognitive perspective on conflict goals management capabilities by proposing an integrative theoretical framework. This framework builds upon the foundational work of Nambisan and Baron (2013), who highlighted the importance of ecosystem-level interactions, and furthers the understanding of how cognitive processes underpin the management of conflict goals. It also addresses the call for deeper inquiry into the “cognition-behavior” dynamics of entrepreneurs, as suggested by the insights of Jia et al. (2023) on the “driver-process-result” perspective.

Secondly, the study offers a processual examination of conflict goals management capabilities, complementing the work of Tushman and Romanelli (1985) who emphasized the need for shifting and transitioning strategies. This research expands upon the antecedents and strategies identified by scholars such as Jarzabkowski and Sillince (2007) by exploring the process mechanisms that connect these elements. The study establishes a complete process mechanism, filling a significant gap in the literature regarding the construction of these capabilities among ecosystem entrepreneurs.

Thirdly, this research enriches the understanding of individual cognitive resources—self-control, grit, and metacognition—in the context of conflict goals management. It acknowledges the contributions of scholars like Duckworth and Quinn (2009) on the role of grit and Wells and Cartwright-Hatton (2004) on metacognition, while also recognizing the need for an integrated approach. This study’s integrative analysis of cognitive dimensions provides a more comprehensive view of their combined effects on entrepreneurial behavior, offering a novel theoretical perspective that can guide future research.

The novelty of this research lies in its holistic approach to understanding the construction of conflict goals management capabilities. By integrating cognitive, processual, and environmental perspectives, this study offers a fresh theoretical lens that captures the complexity of entrepreneurial cognition and behavior within dynamic ecosystems. The research’s focus on the interplay between cognitive resources and the self-regulation process introduces new insights into the adaptive strategies of ecosystem entrepreneurs, setting the stage for a more nuanced understanding of conflict management and entrepreneurial success.

### 5.3 Practical implications

The findings of this study yield actionable insights for ecosystem entrepreneurs, offering a roadmap for enhancing conflict goals management capabilities with practical steps for implementation.

Firstly, it is imperative for ecosystem entrepreneurs to acknowledge that the recognition of conflict goals is paramount to capability development. The interplay between new ventures and the hub firm is subject to trigger factors that can sway the equilibrium of conflict goals. Entrepreneurs must be vigilant in identifying disequilibrium states and proactively address the resulting dependencies or independent actions. A nuanced understanding of these states is essential for strategic decision-making in the face of conflict (Smith and Lewis, 2011).

Secondly, the construction of conflict goals management capabilities should be rooted in cognitive resources and self-regulation. Entrepreneurial cognition theory posits that targeted behaviors stem from underlying cognitive processes. Entrepreneurs are encouraged to leverage cognitive resources and self-regulation as foundational elements in building their management capabilities. This approach aligns with the theoretical framework proposed by Baron and Henry (2010), emphasizing the importance of cognitive effort in entrepreneurial endeavors.

Thirdly, ecosystem entrepreneurs are advised to engage in self-assessment and continuous learning to align with the ecosystem context. Utilizing validated cognitive resource assessment tools can facilitate a thorough evaluation of entrepreneurial skills and readiness. This self-awareness is crucial for gauging adaptability and compatibility within the ecosystem. Furthermore, entrepreneurs can bolster their self-regulation skills through targeted training and education, enabling them to navigate conflict goals with greater finesse and strategic acumen (Feng et al., 2022; Sjödin et al., 2024).

In summary, this study underscores the importance of conflict recognition, cognitive resource utilization, and self-regulation in the practical management of conflict goals within entrepreneurial ecosystems. By providing a structured approach to capability development, it equips entrepreneurs with the tools necessary to thrive in the complex and dynamic ecosystem environment.

### 5.4 Limitations and future work

Some scholars suggest that emotion is also one of the main contents of self-regulation. Future research could consider the role of positive emotion such as passion, optimism, and paradox mindset in the self-regulation process, as well as how cognition and emotion can be organically integrated to jointly construct conflict goals management capabilities. The qualitative research method used in this study, while offering an insightful exploration into the self-regulation process and conflict goals management capabilities, is not without its limitations. The interpretivist approach, which is central to qualitative research, provides a detailed understanding of the phenomena but may not extend to the broader generalizability that quantitative methods can offer. This limitation is a recognized trade-off in qualitative research where depth of analysis is prioritized over breadth. Moreover, this case study cannot quantify the role of cognitive resources in the construction process of conflict goals management capabilities, and it remains unclear whether there is a critical threshold in the effect of cognitive resources, that is, whether an excessive abundance of cognitive resources may lead to negative impacts. In addition, the relationship between cognitive resources and the construction of conflict goals management capabilities identified in this study awaits further empirical research.

## Data availability statement

The original contributions presented in the study are included in the article/supplementary material, further inquiries can be directed to the corresponding author.

## Ethics statement

Ethical approval was not required for the studies involving humans because review and approval were not required for the study on human participants in accordance with the local legislation and institutional requirements. The studies were conducted in accordance with the local legislation and institutional requirements. The participants provided their written informed consent to participate in this study. Written informed consent was obtained from the individual(s) for the publication of any potentially identifiable images or data included in this article.

## Author contributions

WQ: Conceptualization, Data curation, Funding acquisition, Investigation, Methodology, Writing – original draft. SZ: Supervision, Validation, Visualization, Writing – review & editing. BL: Formal analysis, Project administration, Resources, Visualization, Writing – review & editing.
